# Fetal deaths from birth defects in Hunan Province, China, 2016–2020

**DOI:** 10.1038/s41598-024-65985-3

**Published:** 2024-07-02

**Authors:** Xu Zhou, Jian He, Aihua Wang, XinJun Hua, Ting Li, Qin Liu, Junqun Fang, Yurong Jiang, Yingrui Shi

**Affiliations:** Hunan Provincial Maternal and Child Health Care Hospital, Changsha, 410000 Hunan Province China

**Keywords:** Risk factors, Birth defects, Fetal deaths, Stillbirths, Prevalence, Epidemiology, Paediatric research, Medical research, Risk factors

## Abstract

To describe the fetal death rate of birth defects (including a broad range of specific defects) and to explore the relationship between fetal deaths from birth defects and a broad range of demographic characteristics. Data was derived from the birth defects surveillance system in Hunan Province, China, 2016–2020. Fetal death refers to the intrauterine death of a fetus at any time during the pregnancy, including medical termination of pregnancy. Fetal death rate is the number of fetal deaths per 100 births (including live births and fetal deaths) in a specified group (unit: %). The fetal death rate of birth defects with 95% confidence intervals (CI) was calculated by the log-binomial method. Crude odds ratios (ORs) were calculated to examine the relationship between each demographic characteristic and fetal deaths from birth defects. This study included 847,755 births, and 23,420 birth defects were identified. A total of 11,955 fetal deaths from birth defects were identified, with a fetal death rate of 51.05% (95% CI 50.13–51.96). 15.78% (1887 cases) of fetal deaths from birth defects were at a gestational age of < 20 weeks, 59.05% (7059 cases) were at a gestational age of 20–27 weeks, and 25.17% (3009 cases) were at a gestational age of ≥ 28 weeks. Fetal death rate of birth defects was higher in females than in males (OR = 1.25, 95% CI 1.18–1.32), in rural than in urban areas (OR = 1.43, 95% CI 1.36–1.50), in maternal age 20–24 years (OR = 1.35, 95% CI 1.25–1.47), and ≥ 35 years (OR = 1.19, 95% CI 1.11–1.29) compared to maternal age of 25–29 years, in diagnosed by chromosomal analysis than ultrasound (OR = 6.24, 95% CI 5.15–7.55), and lower in multiple births than in singletons (OR = 0.41, 95% CI 0.36–0.47). The fetal death rate of birth defects increased with the number of previous pregnancies (*χ*^*2*^_*trend*_ = 49.28, P < 0.01), and decreased with the number of previous deliveries (*χ*^*2*^_*trend*_ = 4318.91, P < 0.01). Many fetal deaths were associated with birth defects. We found several demographic characteristics associated with fetal deaths from birth defects, which may be related to the severity of the birth defects, economic and medical conditions, and parental attitudes toward birth defects.

## Introduction

Birth defects are structural or functional anomalies at or before birth^1^. The observed prevalence of birth defects is about 2–3% worldwide^2^. Fetal death refers to the intrauterine death of a fetus at any time during the pregnancy, including medical termination of pregnancy (TOP). Fetal death is generally divided into three periods based on gestational age: < 20 weeks of gestation, 20–27 weeks of gestation (early fetal deaths), and ≥ 28 weeks of gestation (late fetal deaths)^3^. Late fetal death is also termed stillbirth^4^. Perinatal deaths include late fetal deaths (or stillbirth) and early neonatal deaths (less than 7 days old)^5^. The stillbirth rate is estimated to be 1.84% globally and 3.55% in China (2015)^6^. Severe birth defects significantly increase the risk of fetal death^7–10^. In developed countries such as Europe and the United States, birth defects have long been the leading cause of perinatal and infant deaths^11^. Therefore, it is of great interest to study fetal deaths from birth defects.

To the best of our knowledge, there is much to add to previous research on birth defects and fetal death. First, although many studies have mentioned that birth defects increase the risk of fetal death, there were fewer studies based on long-term surveillance, especially in China. Second, fewer studies systematically examined the relationship between various specific defects and fetal death. We found the following studies on the association between various specific defects and fetal death. Heinke et al. estimated the risk of stillbirth associated with various specific birth defects in the United States (1997–2011)^9^; Zhou et al. described the relationship between birth defects (including a broad range of specific defects) and perinatal deaths in Hunan Province, China (2010–2020)^10^. Third, few previous studies have addressed the association between early fetal death (< 28 or < 20 weeks of gestation) and birth defects. For example, in China, most studies of birth defects and fetal death have included only fetuses and infants from 28 weeks of gestation to 7 days after birth^10,12^. In several large studies in the United States and Europe, the study population included only birth defects and fetal death at more than 20 weeks of gestation^9,13^. In fact, with the development of prenatal screening and diagnostic methods, an increasing number of birth defects are diagnosed in the first or second trimester of pregnancy^14,15^. Fourth, there are fewer epidemiologic studies on the relationship between birth defects and fetal death.

Therefore, we conducted a retrospective cohort study of fetal deaths from birth defects based on data from the birth defects surveillance system (2016–2020) in Hunan Province, south-central China, to describe the fetal death rate of birth defects (including a broad range of specific defects) and to explore the relationship between fetal deaths from birth defects and a broad range of demographic characteristics.

## Methods

### Data sources

**Figure 1:**
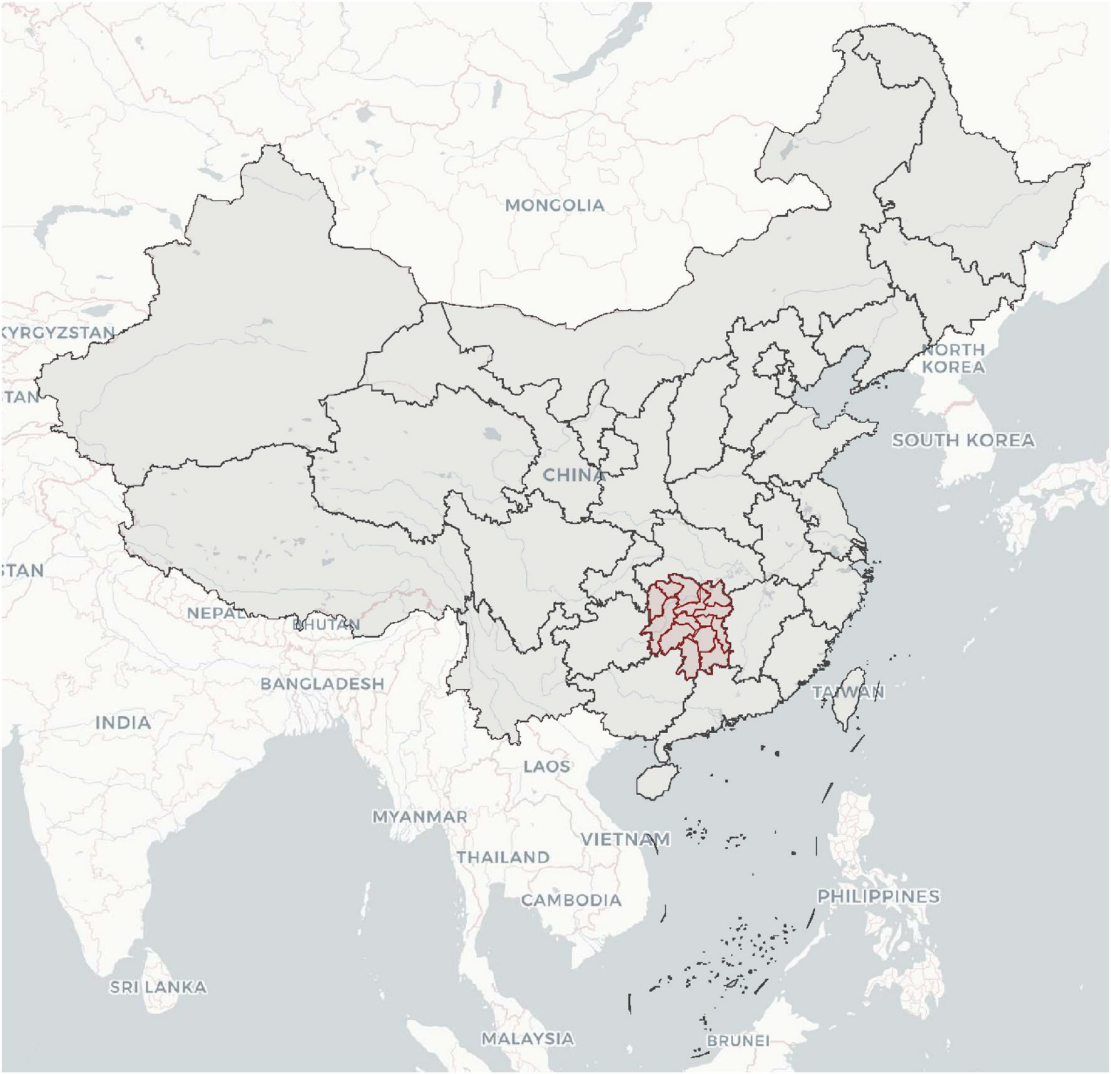
Location of Hunan Province, China.

**Figure 2:**
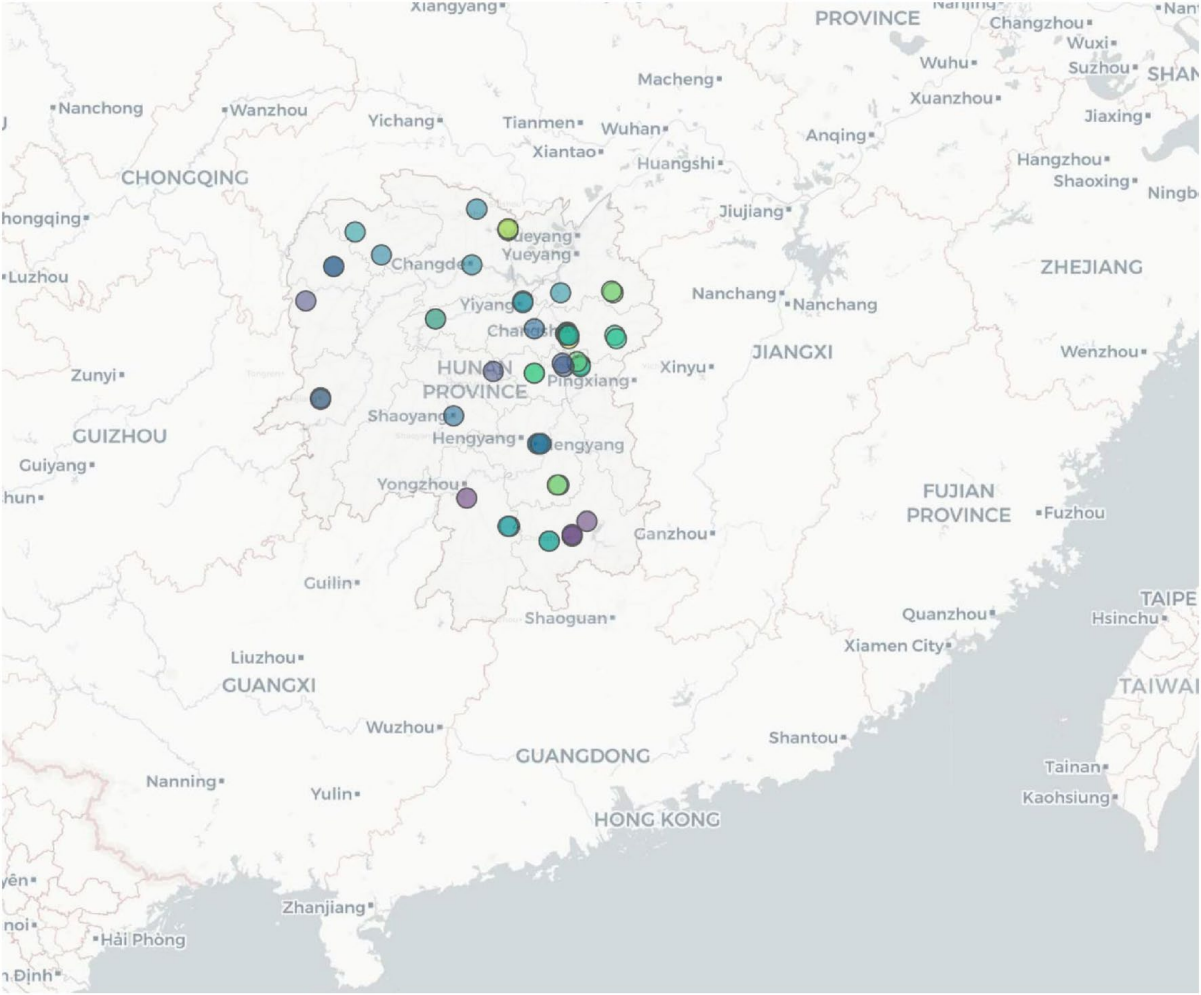
Distribution of birth defects surveillance sites.

This study is a record analysis of hospital-based surveillance. Study data was derived (we accessed the data source on May 25, 2023) from the birth defects surveillance system in Hunan Province, China, 2016–2020, run by the Hunan Provincial Health Commission and involves 52 representative registered hospitals in Hunan Province. In 1996, the Hunan Provincial Health Commission selected those hospitals as surveillance sites, which had undergone a comprehensive evaluation process by experts before the decision. Those 52 hospitals are distributed evenly throughout the province’s municipalities and have well-established services for diagnosing and registering birth defects. Live births in those hospitals account for approximately 1/4 of the total live births in the province. Details are presented in Figs. 1 and 2. Detailed information on birth defects surveillance has been reported elsewhere^12^. The surveillance population included all fetuses born in those hospitals. Surveillance data of fetal deaths (including medical TOP) and birth defects included demographic characteristics such as sex, residence, number of births, maternal age, number of previous pregnancies (including this pregnancy), number of previous deliveries (not including this pregnancy), and diagnostic methods of birth defects (the main methods to identify birth defects).

Birth defects were coded according to the World Health Organization’s International Classification of Diseases (10th edition, ICD-10, codes Q00-Q99). In this study, birth defects were classified into 23 subtypes: anencephaly (Q00), spina bifida (Q05), encephalocele (Q01), hydrocephalus (Q03), cleft palate (Q35), cleft lip (Q36), cleft palate with cleft lip (Q37), microtia/anotia (Q17.2, Q16.0), other external ear defects (Q17), esophageal atresia (Q39), atresia of rectum and anus (Q42), hypospadias (Q54), extrophy bladder (Q64.1), talipes equinovarus (Q66.0), polydactyly (Q69), syndactyly (Q70), limb reduction (Q71, Q72), diaphragmatic hernia (Q79.0), omphalocele (Q79.2), gastroschisis (Q79.3), conjoined twins (Q89.4), Down syndrome (Q90), congenital heart defects (Q20-26) or ‘other’ (Q00-Q99, excluding the above codes).

### Definitions

Fetal death refers to the intrauterine death of a fetus at any time during the pregnancy, including medical TOPs. In this study, any fetal death resulting from a TOP was captured regardless of gestational age, and any fetus with birth defects needs to be certified by a qualified prenatal diagnosis institution before terminating pregnancy. Fetal death is generally divided into three periods based on gestational age: < 20 weeks of gestation, 20–27 weeks of gestation (early fetal deaths), and ≥ 28 weeks of gestation (late fetal deaths). Stillbirth refers to late fetal death (≥ 28 weeks of gestation). Perinatal deaths refer to stillbirths and early neonatal deaths (less than 7 days old). Fetal death rate is the number of fetal deaths per 100 births (including live births and fetal deaths) in a specified group (unit: %). The stillbirth rate is the number of stillbirths per 100 births in a specified group (unit: %). The perinatal mortality rate is the number of perinatal deaths per 100 births in a specified group (unit: %).

### Data quality control

The Hunan Provincial Health Commission developed the “Maternal and Child Health Surveillance Manual in Hunan Province”. Data were collected and reported by experienced and trained doctors and nurses. To ensure data consistency and accuracy, all collectors must be trained and qualified before starting work. The Hunan Provincial Health Commission asks the technical guidance departments to conduct data quality control yearly for all surveillance sites to reduce surveillance data integrity and information error rates.

### Statistical analysis

Prevalence and fetal death rate of birth defects with 95% confidence intervals (CI) were calculated by the log-binomial method^16^. Chi-square trend tests (*χ*^*2*^_*trend*_) were used to determine trends in fetal death rate across the year, number of previous pregnancies, and number of previous deliveries. Chi-square tests (*χ*^*2*^) were used to examine if there were significant differences in fetal death rate by gestational age. Crude odds ratios (ORs) were calculated to examine the relationship between each demographic characteristic and fetal deaths from birth defects.

Statistical analyses were performed using SPSS 18.0 (IBM Corp., NY, USA).

### Ethics approval and consent to participate

The Hunan Provincial Health Commission routinely collected surveillance data, and the government has developed the “Maternal and Child Health Surveillance Manual in Hunan Province” to collect those data. Therefore, there is no additional written informed consent. The Medical Ethics Committee of Hunan Provincial Maternal and Child Health Care Hospital approved the study. (NO: 2023-S012). It is a retrospective study of medical records; all data were fully anonymized before we accessed them. Moreover, we de-identified the patient records before analysis. We confirmed that all operations were following relevant guidelines and regulations.

## Results

### Fetal death rate of birth defects in Hunan Province, China, 2016–2020

This study included 847755 births (including live births and fetal deaths), and 23,420 birth defects were identified, with a prevalence of 2.76% (95% CI 2.73–2.80). A total of 11,955 fetal deaths from birth defects were identified, with a fetal death rate of 51.05% (95% CI 50.13–51.96). From 2016 to 2020, the fetal death rates of birth defects were 50.71%, 47.69%, 51.27%, 52.88%, and 53.73%, respectively, showing an upward trend (*χ*^*2*^_*trend*_ = 23.29, P < 0.01). Among the 11,955 fetal deaths from birth defects, 11811 cases (98.80%) were medical TOP, accounting for 50.43% of all birth defects. (Table 1).

**Table 1:** Fetal death rate of birth defects in Hunan Province, China, 2016–2020.

Year	Births (n)	Birth defects		Fetal deaths from birth defects		Medical termination of pregnancy	
		n	Prevalence (%, 95% CI)	n	Fetal death rate (%, 95% CI)	n	Proportion (%)
2016	170,688	4794	2.81 (2.73–2.89)	2431	50.71 (48.69–52.73)	2365	97.29
2017	196,316	5456	2.78 (2.71–2.85)	2602	47.69 (45.86–49.52)	2564	98.54
2018	177,762	4708	2.65 (2.57–2.72)	2414	51.27 (49.23–53.32)	2387	98.88
2019	164,840	4539	2.75 (2.67–2.83)	2400	52.88 (50.76–54.99)	2400	100.00
2020	138,149	3923	2.84 (2.75–2.93)	2108	53.73 (51.44–56.03)	2095	99.38
Total	847,755	23,420	2.76 (2.73–2.80)	11,955	51.05 (50.13–51.96)	11,811	98.80

*CI* confidence intervals.

15.78% (1887 cases) of fetal deaths from birth defects were at a gestational age of < 20 weeks, 59.05% (7059 cases) were at a gestational age of 20–27 weeks, and 25.17% (3009 cases) were at a gestational age of ≥ 28 weeks. From 2016 to 2020, the number of fetal deaths at a gestational age of < 20 weeks accounted for 12.79%, 13.68%, 15.00%, 19.21%, and 18.83% of all fetal deaths from birth defects, respectively, showing an upward trend (*χ*^*2*^_*trend*_ = 54.92, P < 0.01). Fetal death rates for gestational age < 20 weeks, 20–27 weeks, and ≥ 28 weeks were 100.00%, 99.79%, and 20.81%, respectively, with significant differences (*χ*^*2*^ = 2437.84, P < 0.01). (Table 2).

**Table 2:** Fetal deaths from birth defects according to gestational age.

Year	Gestational age (< 20 weeks)				Gestational age (20–27 weeks)				Gestational age (≥ 28 weeks)			
	Birth defects (n)	Fetal death (n)	Fetal death rate (%)	Proportion among total fetal deaths (%)	Birth defects (n)	Fetal death (n)	Fetal death rate (%)	Proportion among total fetal deaths (%)	Birth defects (n)	Fetal death (n)	Fetal death rate (%)	Proportion among total fetal deaths (%)
2016	311	311	100.00	12.79	1376	1376	100.00	56.60	3107	744	23.95	30.60
2017	356	356	100.00	13.68	1567	1560	99.55	59.95	3533	686	19.42	26.36
2018	362	362	100.00	15.00	1446	1445	99.93	59.86	2900	607	20.93	25.14
2019	461	461	100.00	19.21	1435	1429	99.58	59.54	2643	510	19.30	21.25
2020	397	397	100.00	18.83	1250	1249	99.92	59.25	2276	462	20.30	21.92
Total	1887	1887	100.00	15.78	7074	7059	99.79	59.05	14,459	3009	20.81	25.17

### Fetal death rate of specific defects

The greatest number of fetal deaths occurred among cases with congenital heart defect (3383 fetal deaths, 28.30%), followed by cleft lip with palate (1361 fetal deaths, 11.38%) and Down’s syndrome (1048 fetal deaths, 8.77%). Fetal deaths from these three specific defects accounted for nearly one-half of the total number of fetal deaths from birth defects (48.45%, 5792/11,955). The fetal death rate of conjoined twins was 100%. The fetal death rates of anencephaly (98.75%), encephalocele (98.26%), Down’s syndrome (95.88%), gastroschisis (93.92%), hydrocephalus (93.06%), and cleft lip with palate (91.90%), were higher than 90%. Fetal death rates of spina bifida (87.34%), omphalocele (83.39%), diaphragmatic hernia (78.35%), limb reduction defect (73.36%), extrophy bladder (66.67%), cleft lip (54.78%), esophageal atresia (53.42%), and congenital heart defect (51.33%) ranged from 50 to 80%. Fetal death rates of talipes equinovarus (48.75%), atresia of rectum and anus (21.72%), microtia (14.35%), hypospadias (14.04%), cleft palate (11.15%), syndactyly (8.61%), other defects of external ear (5.81%), and polydactyly (5.39%) were < 50% (Table 3).

**Table 3:** Fetal death rate of specific defects.

Type of birth defect	Specific defects (n)	Prevalence (%, 95% CI)	Fetal deaths (n)	Fetal death rate (%)
Congenital heart defect	6591	0.777 (0.759–0.796)	3383	51.33
Cleft lip with palate	1481	0.175 (0.166–0.184)	1361	91.90
Down’s syndrome	1093	0.129 (0.121–0.137)	1048	95.88
Hydrocephalus	447	0.053 (0.048–0.058)	416	93.06
Talipes equinovarus	802	0.095 (0.088–0.101)	391	48.75
Limb reduction defect	443	0.052 (0.047–0.057)	325	73.36
Omphalocele	319	0.038 (0.033–0.042)	266	83.39
Anencephaly	240	0.028 (0.025–0.032)	237	98.75
Spina bifida	229	0.027 (0.024–0.031)	200	87.34
Cleft lip	345	0.041 (0.036–0.045)	189	54.78
Encephalocele	172	0.020 (0.017–0.023)	169	98.26
Diaphragmatic hernia	194	0.023 (0.020–0.026)	152	78.35
Gastroschisis	148	0.017 (0.015–0.020)	139	93.92
Polydactyly	1968	0.232 (0.222–0.242)	106	5.39
Hypospadias	470	0.055 (0.050–0.060)	66	14.04
Other defects of external ear	1102	0.130 (0.122–0.138)	64	5.81
Syndactyly	662	0.078 (0.072–0.084)	57	8.61
Atresia of rectum and anus	244	0.029 (0.025–0.032)	53	21.72
Esophageal atresia	73	0.009 (0.007–0.011)	39	53.42
Cleft palate	278	0.033 (0.029–0.037)	31	11.15
Microtia	209	0.025 (0.021–0.028)	30	14.35
Conjoined twins	23	0.003 (0.002–0.004)	23	100.00
Extrophy bladder	30	0.004 (0.002–0.005)	20	66.67

*CI* confidence intervals.

Table 4 shows the fetal death rate of specific defects and the proportion of fetal deaths among total fetal deaths according to gestational age (Table 4).

**Table 4:** Fetal death rate of specific defects according to gestational age.

Type of birth defect	Gestational age (< 20 weeks)				Gestational age (20–27 weeks)				Gestational age (≥ 28 weeks)			
	Specific defects (n)	Fetal death (n)	Fetal death rate (%)	Proportion among total fetal deaths (%)	Specific defects (n)	Fetal death (n)	Fetal death rate (%)	Proportion among total fetal deaths (%)	Specific defects (n)	Fetal death (n)	Fetal death rate (%)	Proportion among total fetal deaths (%)
Congenital heart defect	363	363	100.00	10.73	2065	2060	99.76	60.89	4163	960	23.06	28.38
Cleft lip with palate	111	111	100.00	8.16	1108	1106	99.82	81.26	262	144	54.96	10.58
Down's syndrome	116	116	100.00	11.07	836	836	100.00	79.77	141	96	68.09	9.16
Hydrocephalus	65	65	100.00	15.63	184	184	100.00	44.23	198	167	84.34	40.14
Talipes equinovarus	58	58	100.00	14.83	256	255	99.61	65.22	488	78	15.98	19.95
Limb reduction defect	68	68	100.00	20.92	160	157	98.13	48.31	215	100	46.51	30.77
Omphalocele	178	178	100.00	66.92	66	66	100.00	24.81	75	22	29.33	8.27
Anencephaly	165	165	100.00	69.62	58	58	100.00	24.47	17	14	82.35	5.91
Spina bifida	34	34	100.00	17.00	118	118	100.00	59.00	77	48	62.34	24.00
Cleft lip	15	15	100.00	7.94	159	159	100.00	84.13	171	15	8.77	7.94
Encephalocele	77	77	100.00	45.56	78	78	100.00	46.15	17	14	82.35	8.28
Diaphragmatic hernia	17	17	100.00	11.18	96	96	100.00	63.16	81	39	48.15	25.66
Gastroschisis	83	83	100.00	59.71	40	40	100.00	28.78	25	16	64.00	11.51
Polydactyly	17	17	100.00	16.04	63	62	98.41	58.49	1888	27	1.43	25.47
Hypospadias	0	0	–	0.00	12	12	100.00	18.18	458	54	11.79	81.82
Other defects of external ear	0	0	–	0.00	41	41	100.00	64.06	1061	23	2.17	35.94
Syndactyly	7	7	100.00	12.28	29	27	93.10	47.37	626	23	3.67	40.35
Atresia of rectum and anus	0	0	–	0.00	22	22	100.00	41.51	222	31	13.96	58.49
Esophageal atresia	0	0	–	0.00	17	17	100.00	43.59	56	22	39.29	56.41
Cleft palate	4	4	100.00	12.90	20	20	100.00	64.52	254	7	2.76	22.58
Microtia	0	0	–	0.00	24	24	100.00	80.00	185	6	3.24	20.00
Conjoined twins	19	19	100.00	82.61	4	4	100.00	17.39	0	0	–	0.00
Extrophy bladder	3	3	100.00	15.00	7	7	100.00	35.00	20	10	50.00	50.00

### Epidemiology of fetal deaths from birth defects

The fetal death rate of birth defects was higher in females than in males (OR = 1.25, 95% CI 1.18–1.32), in rural than in urban areas (OR = 1.43, 95% CI 1.36–1.50), and lower in multiple births than in singletons (OR = 0.41, 95% CI 0.36–0.47). The fetal death rate of birth defects was higher in maternal age < 20 years (OR = 1.24, 95% CI 1.02–1.52), 20–24 years (OR = 1.35, 95% CI 1.25–1.47), and ≥ 35 years (OR = 1.19, 95% CI 1.11–1.29) compared to maternal age of 25–29 years. Fetal death rate of birth defects was higher in 2 (OR = 1.24, 95% CI 1.16–1.33), 3 (OR = 1.27, 95% CI 1.18–1.36), 4 (OR = 1.27, 95% CI 1.17–1.39), and ≥ 5 (OR = 1.36, 95% CI 1.23–1.49) previous pregnancies compared to first pregnancy. The fetal death rate of birth defects increased with number of previous pregnancies (*χ*^*2*^_*trend*_ = 49.28, P < 0.01). The fetal death rate of birth defects was higher in zero previous delivery compared to one previous delivery (OR = 95.11, 95% CI 67.38–134.26), whereas lower in two previous deliveries (OR = 0.37, 95% CI 0.34–0.39) and ≥ 3 previous deliveries (OR = 0.32, 95%CI: 0.28–0.36) compared to one previous delivery. The fetal death rate of birth defects decreased with the increasing number of previous deliveries (*χ*^*2*^_*trend*_ = 4318.91, P < 0.01). The fetal death rate of birth defects was higher in chromosomal analysis (OR = 6.24, 95% CI 5.15–7.55) and lower in clinical examinations (OR = 0.01, 95% CI 0.00–0.01) compared to ultrasound-diagnosed birth defects (Table 5).

**Table 5:** Epidemiology of fetal deaths from birth defects.

Demographic characteristics	Birth defects (n)	Fetal deaths (n)	Fetal death rate (%)	OR (95% CI)
Sex				
Male	13,291	6127	46.10	Reference
Female	8870	4582	51.66	1.25 (1.18–1.32)
Unknown	1259	1246	98.97	112.07 (64.82–193.76)
Residence				
Urban	11,448	5324	46.51	Reference
Rural	11,972	6631	55.39	1.43 (1.36–1.50)
Number of births				
Singletons	22,337	11,621	52.03	Reference
Multiple births	1083	334	30.84	0.41 (0.36–0.47)
Maternal age (years)				
< 20	409	225	55.01	1.24 (1.02–1.52)
20–24	3105	1771	57.04	1.35 (1.25–1.47)
25–29	9512	4714	49.56	Reference
30–34	6564	3177	48.40	0.95 (0.90–1.02)
≥ 35	3830	2068	53.99	1.19 (1.11–1.29)
Number of previous pregnancies (including this pregnancy)				
1 (First pregnancy)	6562	3067	46.74	Reference
2	6827	3564	52.20	1.24 (1.16–1.33)
3	4802	2528	52.64	1.27 (1.18–1.36)
4	2921	1541	52.76	1.27 (1.17–1.39)
≥ 5	2308	1255	54.38	1.36 (1.23–1.49)
Number of previous deliveries (not including this pregnancy)				
0 (First delivery)	3521	3488	99.06	95.11 (67.38–134.26)
1	11,574	6092	52.64	Reference
2	7215	2086	28.91	0.37 (0.34–0.39)
≥ 3	1110	289	26.04	0.32 (0.28–0.36)
Diagnostic methods of birth defects				
B-Ultrasound	12,924	9445	73.08	Reference
Clinical examinations	5674	91	1.60	0.01 (0.00–0.01)
Chromosomal analysis	2080	1964	94.42	6.24 (5.15–7.55)
Other	2742	455	16.59	0.07 (0.07–0.08)
Total	23,420	11,955	51.05	–

*OR* odds ratio, *CI* confidence intervals.

## Discussion

Overall, many fetal deaths were associated with birth defects, and the fetal death rates of birth defects showed an upward trend from 2016 to 2020. We found that several demographic characteristics were associated with fetal deaths from birth defects. This study is the most recent retrospective cohort study on fetal deaths from birth defects in China and makes some original contributions to the field.

First, the overall fetal death rate of birth defects was relatively high, with significant differences in fetal death rates between specific defects. To our knowledge, the fetal death rate of birth defects (or specific defects) has rarely been reported in previous studies. There were some similar studies. For example, Dolk et al. reported that the fetal death rate of birth defects (gestational age ≥ 20 weeks) was about 20% in Europe (2003–2007)^13^, which was significantly lower than the fetal death rate (51.05%) in this study. It may be mainly associated with better economic and medical conditions in Europe, as better economic and medical conditions are good for the survival of children with birth defects^17^. In this study, the total medical TOP rate of birth defects was 50.43%, and 98.80% of fetal deaths from birth defects were medical TOP. It is rarely reported in previous studies. As far as we know, most medical TOPs were unpreventable deaths because they were very severe birth defects or had severe complications. It may also be partly associated with the relatively poor economic and medical conditions. Zhou et al. reported that the perinatal mortality rate of birth defects (from 28 weeks gestation to 7 days after birth) was 25.03% in Hunan Province, China (2010–2020) ^10^, which was also significantly lower than the fetal death rate in this study. It may be mainly associated with the difference in fetal gestational weeks. As shown in this study, most fetal deaths from birth defects occurred before 28 weeks of gestation. Heinke et al. reported the fetal death rate (gestational age ≥ 20 weeks) of a broad range of specific defects^9^; Zhou et al. also reported the perinatal mortality rate of a broad range of specific defects^10^; Several studies have reported fetal deaths from single specific defects^18–21^. There were also dramatic differences among those studies. It may be mainly associated with differences in prenatal diagnosis rates and medical TOP rates for specific defects.

In addition, in this study, the fetal death rates of birth defects showed an upward trend from 2016 to 2020, which may be mainly related to the fact that an increasing number of fetal deaths from birth defects occurred before 20 weeks of gestation. The underlying reason is the development of prenatal diagnosis techniques, leading to earlier diagnosis of birth defects^22–24^. In general, the earlier the termination of severe birth defects, the lower the adverse effects on the pregnant woman, which leading an increasing number of medical TOP^25,26^. Other findings in this study also supported these conclusions. For example, in this study, the fetal death rate of birth defects diagnosed by chromosomal analysis was the highest, and most chromosomal analyses were prenatal amniocentesis diagnoses at 18–24 weeks of gestation ^27^.

Second, the fetal death rate of birth defects was higher in females than in males. However, previous studies have shown that birth defects occurred more frequently in males than in females^28–30^, including some severe birth defects (e.g., hypospadias, severe congenital heart defects, and congenital anal atresia)^31–33^. It seems to contradict the results of this study. Possible reasons for this phenomenon are the higher prenatal diagnosis rate of birth defects in females than in males and the higher medical TOP rate in females than in males^34^. In addition, the “boy preference” phenomenon exists in some areas of China, especially in poor rural areas^35^, which may also increase the fetal death rate of birth defects in females.

Third, the fetal death rate of birth defects was higher in rural areas than in urban areas, which may be mainly associated with economic and medical conditions. In general, underdeveloped rural areas may lack the medical conditions to treat birth defects or do not have the economic conditions to pay for the treatment^2,36^, which may lead to more fetal deaths from birth defects or prompt parents to choose medical TOP.

Fourth, fetal death rates of birth defects were higher in maternal age < 20 years or ≥ 35 years than in 25–29 years, which may be primarily associated with the severity of the birth defect. For example, previous studies have shown that some serve specific defects were associated with maternal age < 20 years, such as cleft lip and palate^37^, gastroschisis^38^, limb defects^39^, talipes equinovarus^40^, encephaloceles^41^, spina bifida^42^, and anencephaly^43^; and that some serve specific defects were associated with maternal age ≥ 35 years, such as Down syndrome, congenital heart defects, cleft lip with palate, limb reduction, anal atresia, diaphragmatic hernia, omphalocele, and anencephaly^44^.

In addition, some other factors may also contribute to this finding. For example, since most young pregnant women have high reproductive ability and poor economic conditions, they are more likely to terminate fetuses with birth defects and try to conceive healthy babies in the future. Pregnant women of advanced age are in relatively poorer health conditions, making them more likely to suffer from complications during pregnancy, which increases the risk of fetal death^45^. In this study, we also found that the fetal death rate of birth defects was higher in a high number of previous pregnancies and a low number of previous deliveries compared to the reference group. We believe that the mechanism may be similar to that described above. Low maternal age and low number of previous deliveries are similar indicators, reflecting mainly high reproductive ability and partly low economic conditions. Advanced maternal age and the high number of previous pregnancies are also similar indicators, reflecting mainly poorer health conditions. Moreover, the high number of previous pregnancies may be associated with recurrent miscarriages, which may be associated with some severe birth defects^46–48^.

Fifth, the fetal death rate of birth defects was higher in multiple births than in singletons. Some previous studies were consistent with this study. For example, Zhang et al. found that multiple births with birth defects had a higher proportion of live births and early neonatal deaths^49^. However, previous studies have shown that the prevalence of birth defects was higher in multiple births than in singletons. For example, Tang et al. found a higher risk of birth defects in multiple births than in singletons, and the five highest adjusted relative risks for birth defects among multiple births were anencephalus, biliary atresia, hydrocephalus without spina bifida, pulmonary valve atresia and stenosis, and bladder exstrophy, which were severe specific defects^50^. To our knowledge, most multiple births resulted from assisted reproductive technologies, which were associated with many adverse pregnancy outcomes^51,52^ and increased the risk of fetal death from birth defects. The above contradictory findings suggested that the main reason for the lower fetal death rate of birth defects for multiple births may not depend on the prevalence or severity of birth defects. We believe that the fetal death rate of birth defects was higher in multiple births than in singletons, which may be primarily associated with parental attitudes toward birth defects, as pregnant women may find it difficult to get pregnant again.

Some things could be improved in this study. First, the relationship between demographic characteristics and fetal deaths from birth defects may be correlational rather than causal. Second, due to data limitations, some demographic characteristics, such as paternal age and economic conditions, were not included in this study. Third, as mentioned above, fetal deaths may be caused by a variety of diseases, but we did not analyze them in depth due to data limitations. Fourth, many fetuses had multiple birth defects, which may affect the fetal death rate of birth defects. The mechanisms are complex and require in-depth study in the future. Fifth, the surveillance data of this study was derived from some hospitals in Hunan Province, and the surveillance population only accounts for 1/4 of all births, which may not represent the true prevalence of birth defects and fetal death rates. Sixth, there may be some fetuses with minor defects at a gestational age of < 20 weeks that were not ascertained and reported.

## Conclusion

In summary, many fetal deaths were associated with birth defects. We found several demographic characteristics associated with fetal deaths from birth defects, which may be related to the severity of the birth defects, economic and medical conditions, and parental attitudes toward birth defects.

## Data Availability

All data generated or analyzed during this study are included in this published article.
